# Genetic Factors of Autoimmune Diseases 2017

**DOI:** 10.1155/2017/2789242

**Published:** 2017-11-05

**Authors:** Fulvia Ceccarelli, Nancy Agmon-Levin, Carlo Perricone

**Affiliations:** ^1^Lupus Clinic, Reumatologia, Dipartimento di Medicina Interna e Specialità Mediche, Sapienza Università di Roma, Rome, Italy; ^2^Lupus Clinic, Clinical Immunology, Angioedema and Allergy Unit, Zabludowicz Center for Autoimmune Diseases, Sheba Medical Center, Ramat Gan, Israel

Autoimmune diseases are characterized by a multifactorial aetiology, in which genetic factors interplay with environmental factors. The different genetic factors are associated not only with disease susceptibility but also with specific autoantibodies and disease phenotypes.

Several studies have been conducted on this issue, identifying different genetic loci suspected to be involved in systemic autoimmune disease pathogenesis. Interestingly, autoimmune diseases share several risk loci, suggesting the involvement of common pathways to loss of tolerance.

Genetic factors associated with rheumatoid arthritis (RA) development have been widely investigated. Of these, the human leukocyte antigen (HLA) region contributes to approximately half of its genetic susceptibility, particularly in disease characterized by the presence of anti-citrullinated antibodies. Notably, a number of alleles in the epitope recognition part of the HLA molecule which is strongly associated with RA share a common string of amino acid residues, the so-called shared epitope. Next to HLA genes, other variants seem to be implicated in RA susceptibility such as the PTPN22, TRAF1-C5, PADI4, and STAT4 genes. Moreover, genetic factors seem to contribute to a disease phenotype, especially in terms of erosive damage. Recent data suggest an association between radiographic damage and polymorphisms of genes encoding TNF, IL-1, IL-6, IL-4, IL-5, OPN, and PRF1.

Considering systemic lupus erythematosus (SLE) genetic variabilities which were identified so far, it has been demonstrated that the latter accounts for less than half of this disease heritability, by the modest overall effect sizes. In this context, HLA loci as well as non-HLA risk loci (i.e., STAT4, PTPN22, IFIH1, and TRAF3IP2) have been associated with SLE susceptibility. Moreover, the disease heterogeneity in terms of clinical manifestations and outcomes has been demonstrated to be associated with specific genetic factors leading to the protean clinical picture. These evidences allow the possibility of elucidating different mechanisms and pathways accountable for disease manifestations. However, except for lupus nephritis associations with ITGAM and IRF gene polymorphisms, no studies have been designed to identify the genetic variants associated with the development of different SLE phenotypes.

In the last years, some studies investigated the genetic links to other autoimmune diseases, especially organ-specific ones. Among these, some data are available herein regarding the genetic background of primary biliary cholangitis (PBC), an autoimmune cholestatic liver disease, characterized by the antimitochondrial autoantibody positivity and by the accumulation of antigen-specific autoreactive B and T cells targeting biliary epithelial cells. The aetiology of PBC has yet to be fully defined; however, similar to other autoimmune diseases, the human leukocyte antigen (HLA) class II alleles have been significantly associated with disease susceptibility. More recently, genome-wide association studies have identified also non-HLA genes implicated in PBC development. Of these, the strongest associations were identified for genetic variants of IL12A and IL12RB2 genes, but other polymorphisms, such as STAT4 and CTLA4, have been also recognized. The recognition of specific genetic variants may be helpful in the elucidation of pathogenic mechanisms underlying PBC development as well as in the identification of patients with a more aggressive disease course.

Another angle to the *scenario* of genetics and autoimmunity is related to the role of virus-derived microRNA in the genome stability. As widely demonstrated, infections play a crucial role as environmental factors affecting the development of autoimmune diseases. Viral infections, through noncoding RNAs termed microRNA, could interplay with the host genome, allowing modifications that could be responsible for a dysregulation of an immune response, as was reported for the Epstein-Barr virus.

In conclusion, in the last years, the focus of genetic studies shifted towards other autoimmune diseases and environmental factors that may modify the host genome. These new aspects of the mosaic between the gene environment and diseases may enable us to better understand the pathogenic mechanisms responsible for the loss of tolerance and the development of immune-related diseases.

*Fulvia Ceccarelli* *Nancy Agmon-Levin* *Carlo Perricone*

